# 

**Published:** 1869-11

**Authors:** 


### Books and Pamphlets Received.

A Compend of Materia Medica and Therapeutics. For the use of Students. By John C. Riley, A. M., M. D., Professor of Materia Medica and Therapeutics in the National Medical College; one of the Physicians of Providence Hospital, Washington, D. C., etc.

Transactions of the Twenty-Fourth Annual Meeting of the Ohio State Medical Society, held at Columbus, June 8th, 9th and 10th, 1869.

History of Four Cases of Chronic Inversion of the Uterus; with the account of an Operation designed as a substitute for Amputation. By T. Gaillard Thomas, M. D., New York.

First Annual Announcement of the Kansas City College of Physicians and Surgeons, Kansas City. Mo.

Aiken; or, Climatic Cure. By Amory Coffin, M. D.. and W. H. Geddings, M.D.

Catalogue of the Museum and Library of the Hahnemann Medical College of Philadelphia.

Pepsin: Its Physiological and Therapeutical Actions. By J. S. Hawley, A M., M. D.

Bossange's Catalogue of Periodicals. An abridged list of the principal French papers and serials, with prices of subscription for Paris and the United States.—Gustave Bossange, 25 Quai Voltaire, Paris.

